# Relapse patterns and radiation dose exposure in IDH wild-type glioblastoma at first radiographic recurrence following chemoradiation

**DOI:** 10.1007/s11060-022-04123-3

**Published:** 2022-09-02

**Authors:** Satoka Shidoh, Ricky R. Savjani, Nicholas S. Cho, Henrik E. Ullman, Akifumi Hagiwara, Catalina Raymond, Albert Lai, Phionah L. Nghiemphu, Linda M. Liau, Whitney B. Pope, Timothy F. Cloughesy, Tania B. Kaprealian, Noriko Salamon, Benjamin M. Ellingson

**Affiliations:** 1 UCLA Brain Tumor Imaging Laboratory (BTIL), Center for Computer Vision and Imaging Biomarkers, Department of Radiological Sciences, David Geffen School of Medicine, University of California Los Angeles, 924 Westwood Blvd., Suite 615, Los Angeles, CA 90024 USA; 2 Department of Radiological Sciences, David Geffen School of Medicine, University of California Los Angeles, Los Angeles, CA USA; 3 Departmet of Neurosurgery, Washington University in St. Louis, St. Louis, MO USA; 4 Department of Radiation Oncology, David Geffen School of Medicine, University of California Los Angeles, Los Angeles, CA USA; 5 Medical Scientist Training Program, David Geffen School of Medicine, University of California, Los Angeles, Los Angeles, CA USA; 6 Department of Bioengineering, Henry Samueli School of Engineering and Applied Science, University of California Los Angeles, Los Angeles, CA USA; 7 Department of Radiology, Juntendo University School of Medicine, Tokyo, Japan; 8 Department of Neurology, David Geffen School of Medicine, University of California Los Angeles, Los Angeles, CA USA; 9 Department of Neurosurgery, David Geffen School of Medicine, University of California Los Angeles, Los Angeles, CA USA; 10 Department of Psychiatry and Biobehavioral Sciences, David Geffen School of Medicine, University of California Los Angeles, Los Angeles, CA USA; 11 Department of Radiology, Washington University School of Medicine, St. Louis, MO USA

**Keywords:** Glioblastoma, Radiation therapy, Patterns of progression

## Abstract

**Purpose:**

To quantify the radiation dose distribution and lesion morphometry (shape) at baseline, prior to chemoradiation, and at the time of radiographic recurrence in patients with glioblastoma (GBM).

**Methods:**

The IMRT dose distribution, location of the center of mass, sphericity, and solidity of the contrast enhancing tumor at baseline and the time of tumor recurrence was quantified in 48 IDH wild-type GBM who underwent postoperative IMRT (2 Gy daily for total of 60 Gy) with concomitant and adjuvant temozolomide.

**Results:**

Average radiation dose within enhancing tumor at baseline and recurrence was ≥ 60 Gy. Centroid location of the enhancing tumor shifted an average of 11.3 mm at the time of recurrence with respect to pre-IMRT location. A positive correlation was observed between change in centroid location and PFS in MGMT methylated patients (*P* = *0.0007*) and Cox multivariate regression confirmed centroid distance from baseline was associated with PFS when accounting for clinical factors (*P* = *0.0189*). Lesion solidity was higher at recurrence compared to baseline (*P* = *0.0118*). Tumors that progressed > 12 weeks after IMRT were significantly more spherical (*P* = *0.0094*).

**Conclusion:**

Most GBMs recur local within therapeutic IMRT doses; however, tumors with longer PFS occurred further from the original tumor location and were more solid and/or nodular.

## Introduction

Glioblastoma (GBM) remains the most aggressive and fatal form of primary brain tumor in adults with an extremely poor prognosis [1]. Standard of care for newly diagnosed GBM includes maximal safe surgical resection followed by postoperative radiation therapy with concurrent temozolomide chemotherapy [2] (with or without tumor treating fields [3]). However, recurrence rates of GBM are ~ 90%, and there remains no standard of care for recurrent GBM [4]. Repeat surgery can only be considered for ~ 25% of patients of recurrent GBM because of concerns of post-operative morbidity, and despite advances in reirradiation techniques for accurate target delineation, neurological toxicity from radiation-induced brain necrosis remains a considerable risk [4, 5]. While previous studies have shown that dose escalation at standard fractionation (1.8–2 Gy daily) up to 60 Gy improves survival [6, 7], tumor recurrence most often occurs locally within the original 95% isodose zone [8–10]. Therefore, there is a need for a contemporary and detailed analysis quantifying the incidence and degree of local recurrence, as well as how these characteristics correspond with patterns of progression in newly diagnosed GBM.

Recently, there has also been growing interest in exploring the morphological features of GBM on MRI. For example, metrics such as sphericity and solidity have been explored to characterize newly diagnosed GBM subtypes and offer prognostic value [11–13]. However, to our knowledge, there has been little morphological analysis of GBM at radiologic recurrence, and the analyses remains limited to qualitative descriptors [14]. Because recurrent GBMs are known to significantly differ from the primary tumor in terms of gene mutations, gene expression, and tumor microenvironment [15, 16], along with evidence that specifically chemoradiation may have a role in the altered phenotype of recurrent GBMs [16–18], a longitudinal assessment of GBM recurrence involving radiation dose distribution, morphological metrics, and molecular subtypes may provide insights into the alterations that occur in recurrent GBMs and their associated factors.

In the current study, we explored the dose distribution within enhancing tumor and lesion morphological metrics at baseline and at the time of recurrence. Based on previous observations, we hypothesized that the vast majority of recurrent tumors would recur locally within therapeutic radiation doses and that tumor recurrence at longer progression free survival times would be more solid and/or nodular.

## Materials and methods

### Patient characteristics

A total of 48 patients with histologically confirmed IDH1 wild-type GBM who underwent postoperative intensity-modulated radiotherapy (IMRT) (2 Gy daily, total 60 Gy) with concomitant temozolomide (75 mg/m^2^/day, 7 days per week during radiotherapy, followed by 1 month break) and 6–12 cycles of adjuvant chemotherapy at 150 mg/m^2^/day to 200 mg/m^2^/day between 2011 and 2019 were included in the current study. Patients were included in the study if they had high quality MRI data and radiation planning data available for evaluation prior to and through the point of radiographic progression according to the standard RANO criteria [19]. If patients progressed within 3 months of completion of chemoradiation, a modified RANO criteria [20] was used and progression was confirmed by a subsequent scan exhibiting progressive enhancement in order to limit pseudoprogression. Patients were not included if they were treated with upfront experimental therapies including bevacizumab. All scans were evaluated twice, once using bidirectional measurements to determine the time to progression via RANO and once by contouring the entire lesion for a volumetric assessment and subsequent analyses. All scans were retrospectively evaluated and measured by a board-certified neurosurgeon (SS with 15 years of clinical experience) and reviewed by fellowship trained neuroradiologists (H.E.U., A.H., N.S.). All patients provided informed written consent to be included in our IRB approved Neuro-Oncology database.

### Radiation treatment planning

**Fig. 1:**
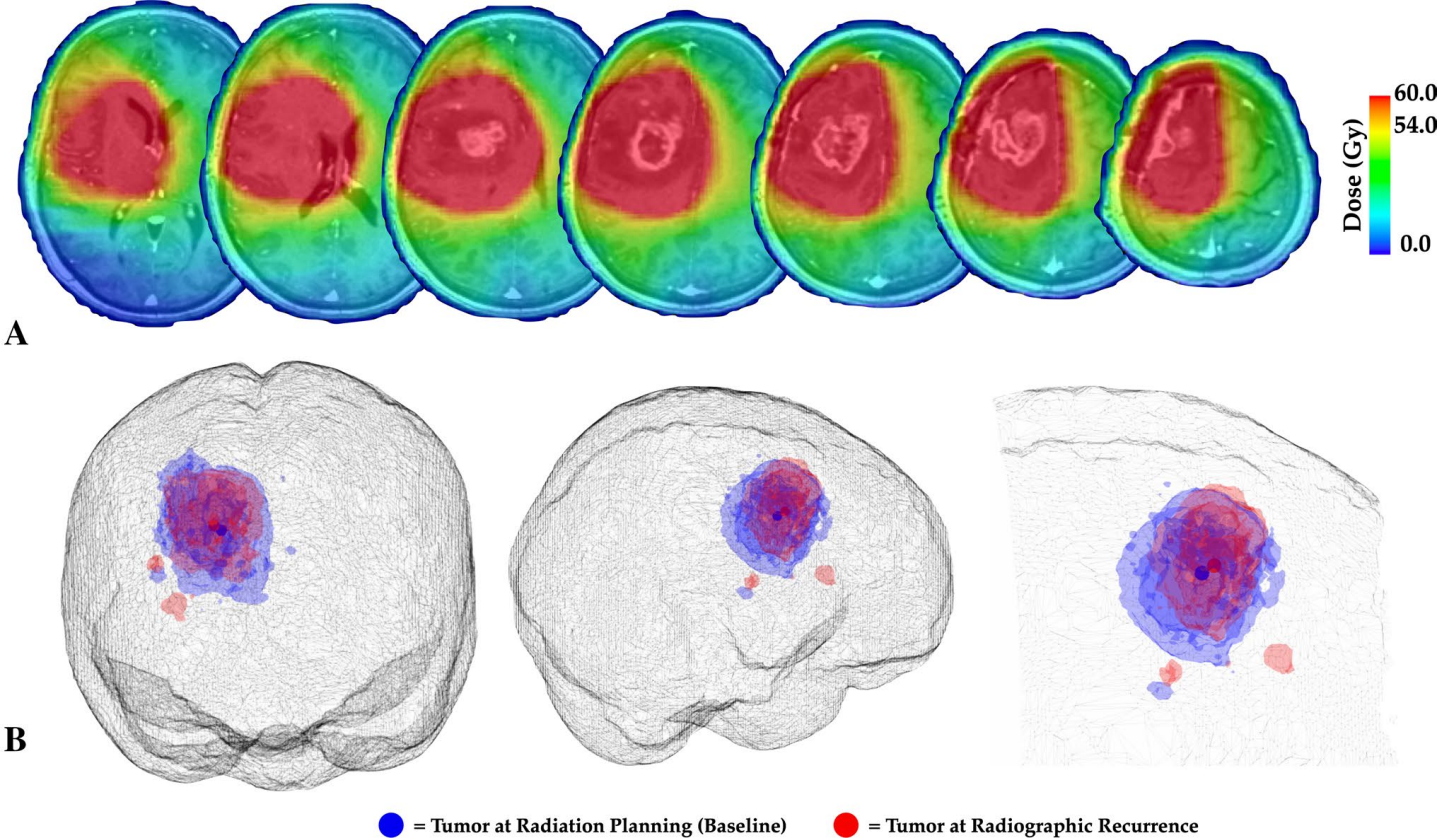
**A** Fusion of radiation treatment dose planning with pre-radiation MRI scans. **B** Segmented enhancing tumor at baseline (blue) and at the time of radiographic recurrence (red) as well as the centroid location for both (solid spheres in center of enhancing regions of interest)

Radiation treatment planning was performed as part of clinical standard of care by a radiation oncologist specialized in treatment of central nervous system disorders. Contours were drawn on MIM (MIM Vista, Cleveland, Ohio). Treatment planning was performed on the Eclipse platform (Varian Medical Systems, Palo Alto, CA). Treatment planning was made using CT image data, then rigidly co-registered (see details below) to contrast enhanced T1-weighted, T2-weighted and T2-weighted fluid attenuated inversion recovery (FLAIR) sequences (Fig. 1A). The gross tumor volume (GTV) for radiation planning was defined as the enhanced lesion at postoperative contrast-enhanced MRI. The clinical target volume (CTV) consisted of GTV lesion plus a 2–3 cm margin.

### MRI acquisition and post-processing

Anatomic MRI consisted of pre- and post-contrast axial T1 weighted images acquired using either 2D using a turbo spin echo (TSE) acquisition with 3 mm slice thickness and no interslice gap or a 3D inversion prepared gradient echo (IR-GRE) acquisition with 1–1.5 mm isotropic voxel size according to the international standardized brain tumor imaging protocol (BTIP) [21]. T2-weighted TSE and T2-weighted FLAIR images were acquired with 3 mm slice thickness and no interslice gap according to BTIP recommendations. All images and radiation planning fields were registered to the Montreal Neurological Institute (MNI) space using a 12-degree of freedom-transformation with a mutual information cost function and a tri-linear interpolation (FLIRT, http://www.fmrib.ox.ac.uk/fsl/; FSL Version 6.0). 3D regions of interests (ROIs) constituting contrast enhancing tumor were segmented based on the region of hyperintensity in contrast enhanced T1-weighted digital subtraction maps, using a semiautomatic procedure and the Analysis of Functional Neuro-Images (AFNI) software (NIMH Scientific and Statistical Computing Core: Bethesda, MD, USA) as described previously [22].

### Estimation of recurrence patterns and radiation dose exposure

In order to determine whether tumors were more likely to progress locally near the site of the original tumor bed or distal from the original treated tumor, the center of mass of the enhancing tumor at baseline and at the time of tumor recurrence was calculated using custom scripts in *Python* (https://python.org) and *Scipy* (https://scipy.org), then the distance between the centroid location of the original tumor and recurrence was estimated from these coordinates (Fig. 1B). We also calculated tumor sphericity, or how spherical the tumor is, and solidity [11, 23], or how irregular or dense (non-porous) the enhancing component of the tumor is, as defined by:

1 $$ Sphericity = \Psi = \frac{{{\pi^{1/3}}{{(6V)}^{2/3}}}}{A}$$

2 $$ Solodity = \Theta = \frac{V}{Convex\;Volume}$$

where *V* is the enhancing tumor volume, *A* is the enhancing tumor surface area, and *Convex Volume* refers to the volume of an object defined by the convex hull of the object. Lastly, the radiation dose distribution to the original tumor bed and the enhancing tumor at the time of recurrence was calculated after fusion of radiation fields with the contoured tumor regions of interest.

### Hypothesis testing and statistical analysis

Based on the current literature, we theorized that most tumors would recur near the site of the original treated tumor [24]. We hypothesized the centroid of the enhancing tumor at the time of radiographic recurrence would be ≤ 5 mm of the pre-treatment tumor centroid location based on a study by Tu et al*.* that observed most GBMs recurred within 5-mm of the contrast-enhanced tumor and T2/FLAIR tumor boundaries [24]. To test this, we performed a one-sided t-test to determine whether the mean centroid distance was greater than 5 mm. We also hypothesized that tumors that recur at a later time point, having longer progression free survival (PFS), would be more distal from the original tumor location and perhaps outside the original radiation field, particularly in MGMT promoter methylated patients. To test these hypotheses, we performed linear regression and used Pearson’s correlation coefficient for all patients and MGMT subtypes and tested whether the slope and/or intercept of this correlation differed from zero. We also used Cox multivariable regression to further explore the association between PFS, overall survival (OS), or post-progression survival (PPS) and MGMT status or distance between centroids at recurrence. Statistical analyses were performed with *R* software (version 4.0.3; http://www.r-project.org/) and GraphPad Prism (Version 8.0c; GraphPad Software, La Jolla, Ca, USA). Statistical significance was defined as P < 0.05, with no multiple comparison corrections, and all tests were two-tailed.

## Results

Of the 48 patients enrolled in the current study, 19 were female and 29 were male. The average age of the participants was 56.2 years old (± 10.4 s.d.) and the average KPS at baseline was 83 and the range was 50 to 90. A total of 18 patients were MGMT methylated (38%) and 28 patients were unmethylated (58%), with 2 having unknown MGMT status due to insufficient quantity of tissue for testing. Mean volume of the baseline tumor was 12.36 mL (range 0.01–61.13 mL) and the average enhancing tumor volume was 9.19 mL (range 0.44–86.10 mL) after completion of radiotherapy. At the time of recurrence via RANO, average enhancing tumor volume for the cohort was 8.46 mL (range 0.13–86.10 mL). This was likely due to 10 of the 48 patients (20.8%) exhibiting tumor shrinkage before the time of radiographic recurrence (1 of these 10 were MGMT promoter methylated) and another 15 of the 48 patients (31.3%) exhibiting tumor recurrence due to an unmeasurable new lesion. The median OS for the final cohort was 30.5 months, the median PFS was 4.0 months, and the median post-progression survival (PPS) was 19.6 months. Additional patient characteristics are shown in Table 1.

**Table 1:** Patient demographics

Number of patients	48
Age [years] (range)	56.2 (32–73)
Sex	
Male (N)	58% (28)
Female (N)	42% (20)
Karnofsky performance score (KPS)	83.3 (50–90)
MGMT status	
Methylated	37.5% (18)
Unmethylated	58.3% (28)
No result	4.2% (2)

### Patterns of recurrence

**Fig. 2:**
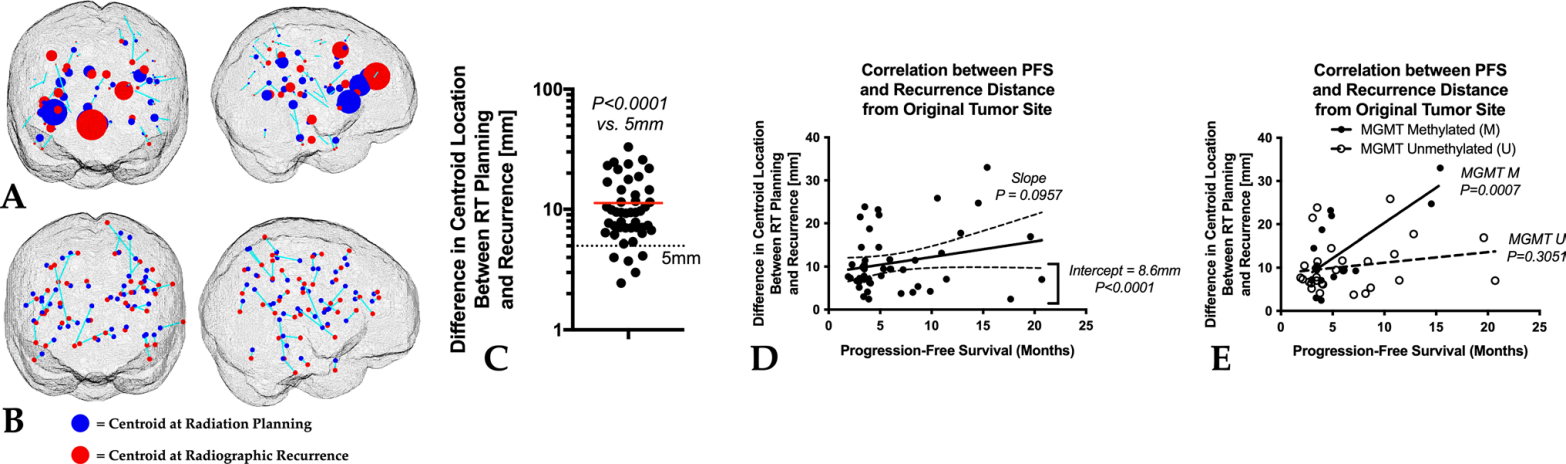
Association between tumor centroid location and outcomes. **A** Centroid location for all patients in the current study, where size of the spheres represents relative (normalized) tumor volume, for baseline (blue, radiation planning) and at the time of radiographic recurrence (red). **B** Centroid location for all patients in the study with uniform sphere size, meant to illustrate similar centroid distances among patients (size of blue line between the red and blue spheres). **C** Difference between centroid location at baseline or planning compared with centroid location at the time of radiographic recurrence, showing more than 5 mm shift in tumor location at the time of recurrence (*P* < *0.0001*). **D** Correlation between progression-free survival (PFS) and difference in centroid location between baseline and recurrence for all patients and **E** for patients separated by MGMT status. *MGMT M* MGMT methylated, *MGMT U* MGMT unmethylated

As depicted in Fig. 2A, B, the centroid of the enhancing tumor shifted an average of 11.3 mm (± 1.03 S.E.M.) at the time of radiographic recurrence relative to the baseline, pre-RT planning scan. This was significantly greater than the theorized limit of 5 mm from the original site (Fig. 2C; *one-sided t-test, P* < *0.0001*). A positive trend was observed between difference in tumor centroid location and PFS when pooling all patients (Fig. 2D; *P* = *0.0957*) and a significant bias was observed in this correlation (intercept = 8.6 mm; *P* < *0.0001*), suggesting an average change in centroid distance of around 8.6 mm regardless of PFS. When patients were stratified based on MGMT status, a significant positive correlation was observed between centroid differences and PFS in MGMT methylated (Fig. 2D; *P* = *0.0007*) but not unmethylated (*P* = *0.3051*) GBM patients. Cox multivariable regression confirmed that continuous measures of centroid distance was associated with PFS (*P* = *0.0189, HR* = *0.9481*), while MGMT status (*P* = *0.0705*) was not significantly associated with PFS. No significant association was observed between centroid distance and post-progression survival (PPS), defined as the difference between the time of radiographic progression to death (*Cox, P* = *0.6828*).

**Fig. 3:**
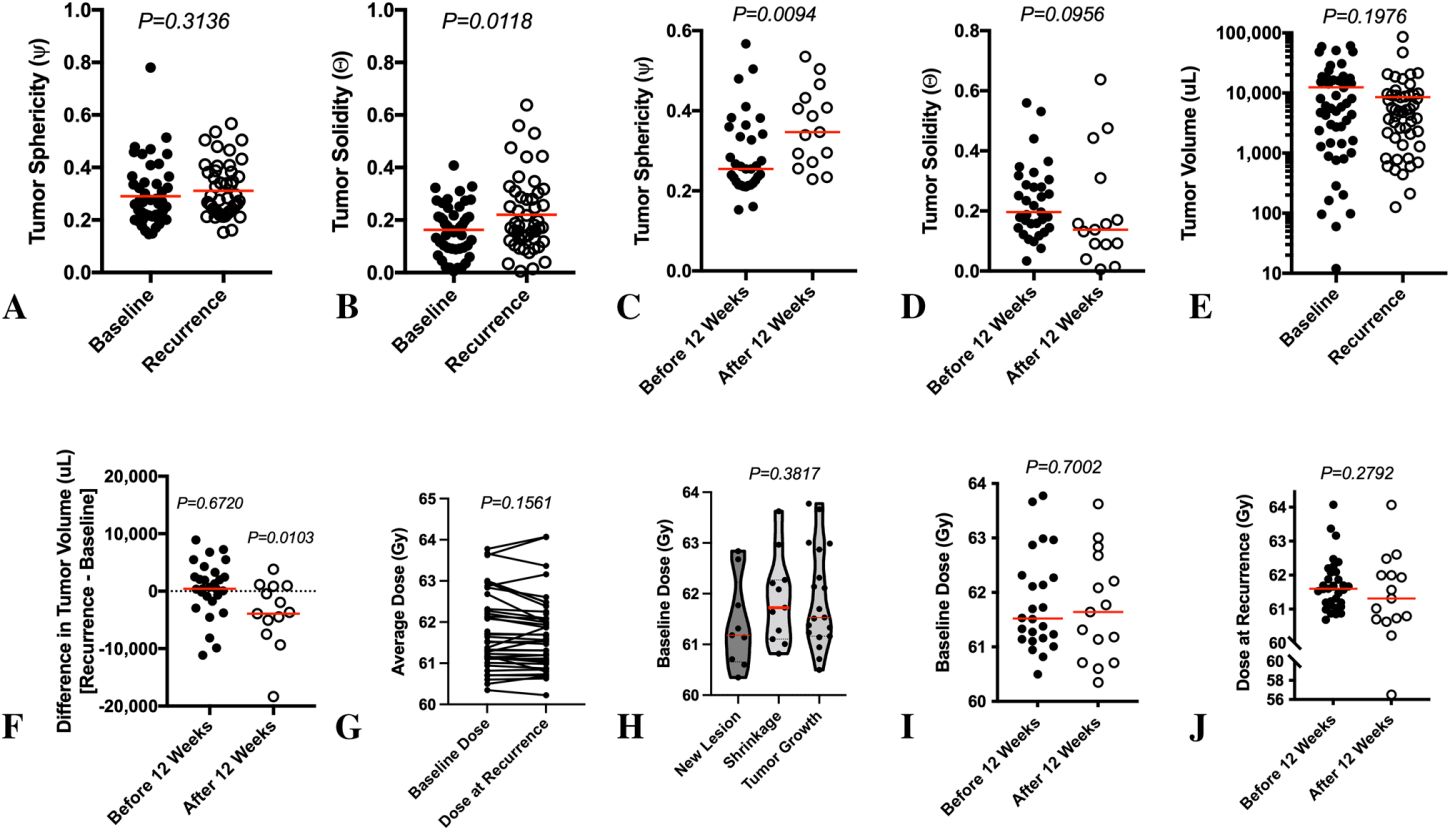
**A** Tumor sphericity and **B** solidity of enhancing tumor at baseline (treatment planning) and radiographic recurrence. **C** Tumor sphericity and **D** solidity at the time of recurrence for patients exhibiting radiographic progression before and after 12 weeks following completion of chemoradiation. **E** Tumor volume at baseline and at the time of radiographic recurrence. **F** Difference in tumor volume at the time of radiographic recurrence with respect to pre-treatment baseline for patients exhibiting radiographic progression before and after 12 weeks following completion of chemoradiation. **G** Average tumor dose for each patient within the enhancing tumor at baseline and at the time of radiographic recurrence after alignment to pre-treatment image space. **H** Radiation dose within the enhancing tumor at baseline for patients who exhibited new lesions, tumor shrinkage, and tumor growth in response to chemoradiation treatment. **I** Baseline, pre-treatment radiation dose and **J** dose at the time of radiographic recurrence within the enhancing tumor compared between patients exhibiting radiographic progression before and after 12 weeks following completion of chemoradiation

No difference in tumor sphericity was observed between baseline, pre-radiation scans, and the scans at radiographic progression (Fig. 3A; *paired t-test, P* = *0.3136*); however, a significantly higher level of solidity was observed at the time of tumor recurrence relative to the pre-radiation baseline time point (Fig. 3B; *P* = *0.0118*). At recurrence, enhancing tumors were significantly more spherical in shape if they progressed after 12 weeks (Fig. 3C; *Mann–Whitney, P* = *0.0094*) and trended toward lower solidity (Fig. 3D; *Mann–Whitney, P* = *0.0956*). Neither sphericity (*Cox, P* = *0.2924*) nor solidity (*Cox, P* = *0.7462*) were independent predictors of PPS.

Of the 48 patients enrolled in the current study, 15 patients had recurrence by a new measurable enhancing lesion (31%) and 10 patients had tumor shrinkage prior to radiographic progression (21%), so the time to progression for these patients was determined by the nadir. On average for all patients (Fig. 3E), mean tumor volume prior to radiation treatment was 12.4 mL (median = 5.3 mL) and mean tumor volume at the time of recurrence was 8.5 mL (median = 4.6 mL). In patients exhibiting new enhancing lesions or shrinking tumors, the average tumor volume at baseline was 13.7 mL and 12.5 mL, while the average tumor volume was 7.0 mL and 2.8 mL at recurrence, respectively. For patients without evidence of new lesions or evidence of tumor shrinkage (48%), tumor volume at baseline was 10.9 mL and 11.1 mL at recurrence. There was no significant difference in tumor volume between baseline and recurrence for patients with radiographic progression prior to 12 weeks (Fig. 3F; *P* = *0.1976*), but patients who progressed after 12 weeks from the start of radiation demonstrated a significant reduction in tumor volume compared to baseline (*P* = *0.0103*).

Cox univariate survival analysis showed no significant association between continuous measures of pre-radiation enhancing tumor volume (*P* = *0.6020*), post-radiation enhancing tumor volume (*P* = *0.7945*), or the difference in enhancing tumor volume between pre- and post-radiation time points and PFS (*P* = *0.3415*). Similarly, no significant associations were observed between continuous measures of pre-radiation enhancing tumor volume (*P* = *0.2700*), post-radiation enhancing tumor volume (*P* = *0.9808*), or the difference in enhancing tumor pre- and post-radiation and OS (*P* = *0.2445*). Enhancing tumor volume at the time of radiographic recurrence was not associated with PPS (*P* = *0.6478*). Cox multivariate analysis including age and MGMT status as covariates showed comparable lack of significance between all enhancing tumor volume measurements and PFS, OS, and PPS.

### Effects of radiation dose

**Fig. 4:**
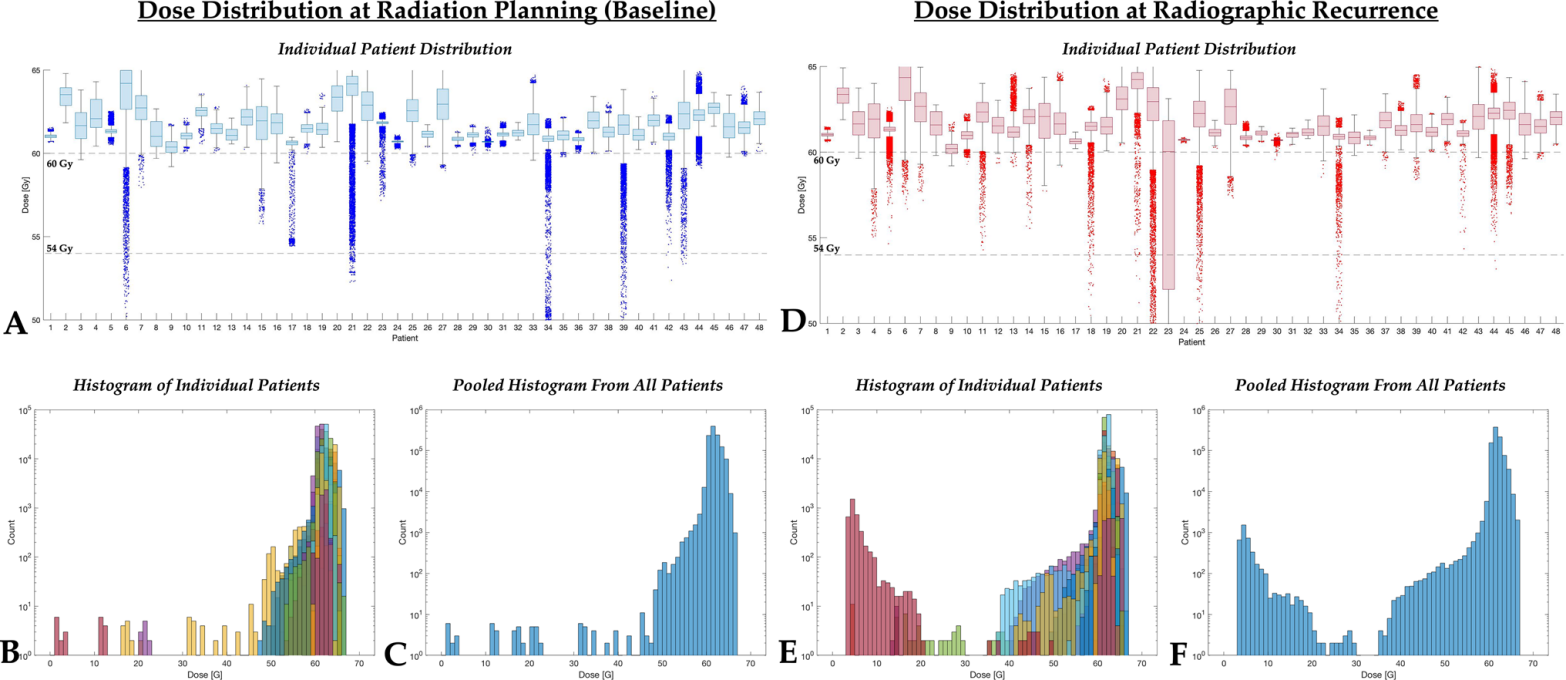
Voxel-based dose distribution for individual patients. **A** Voxel-wise dose distribution within enhancing tumor at baseline or radiation planning. **B** Log_10_-transformed histogram of voxel-wise dose distribution individual patients, color coded by individual patients. **C** Log_10_-transformed histogram of voxel-wise dose distribution pooled for all patients. **D** Voxel-wise dose distribution within enhancing tumor at the time of radiographic recurrence after alignment to pre-treatment image space and fused with radiation dose prescriptions. **E** Log_10_-transformed histogram of voxel-wise dose distributions at the time of radiographic recurrence, color coded by individual patients. **F** Log_10_-transformed histogram of voxel-wise dose distributions at the time of radiographic recurrence, pooled across all patients

The average radiation dose to the enhancing tumor across all patients at the pre-radiation baseline time point was 61.8 Gy (median = 61.5 Gy, range = 60.3–63.8 Gy) and the average radiation dose exposed by the enhancing tumor at the time of recurrence was 61.6 Gy (median = 61.6 Gy, range = 56.5–64.1 Gy). No difference in the average radiation dose within enhancing tumor at baseline and the average radiation dose within the enhancing tumor at recurrence was identified (Fig. 3G; *P* = *0.1561*). No significant difference in the average dose within the enhancing tumor at baseline compared with the pattern of recurrence was observed (Fig. 3H; *P* = *0.3817*), although patients exhibiting tumor shrinkage prior to tumor recurrence had an average radiation dose of ~ 0.5 Gy higher than the average dose to the enhancing tumor at baseline in patients who exhibited new, non-measurable enhancing lesions at the time of radiographic progression. No difference in baseline radiation dose (Fig. 3I; *P* = *0.7002*) or dose at recurrence (Fig. 3J; *P* = *0.2792*) was observed between patients who progressed before or after 12 weeks following radiation therapy. Additionally, no significant associations were observed between continuous measures of average radiation dose within the enhancing tumor at baseline and PFS (*Cox, P* = *0.2045*) or OS (*P* = *0.7907*), even after accounting for both age and MGMT status (*P* = *0.2047* for PFS and *P* = *0.8626* and OS). Similarly, no association was observed between the average radiation dose prescribed at baseline to the site of future GBM recurrence and the time of radiographic recurrence and PPS (*P* = *0.7583*) even after controlling for age and MGMT status (*P* = *0.8412*). However, when examining voxel-based dose distributions for individual patients (Fig. 4), the distribution of dose within the enhancing tumor at both the baseline time point and at radiographic recurrence was quite variable. For example, at recurrence, there was a larger proportion of enhancing tumor voxels having received a reduced dose of ≤ 40 Gy than at baseline.

**Fig. 5:**
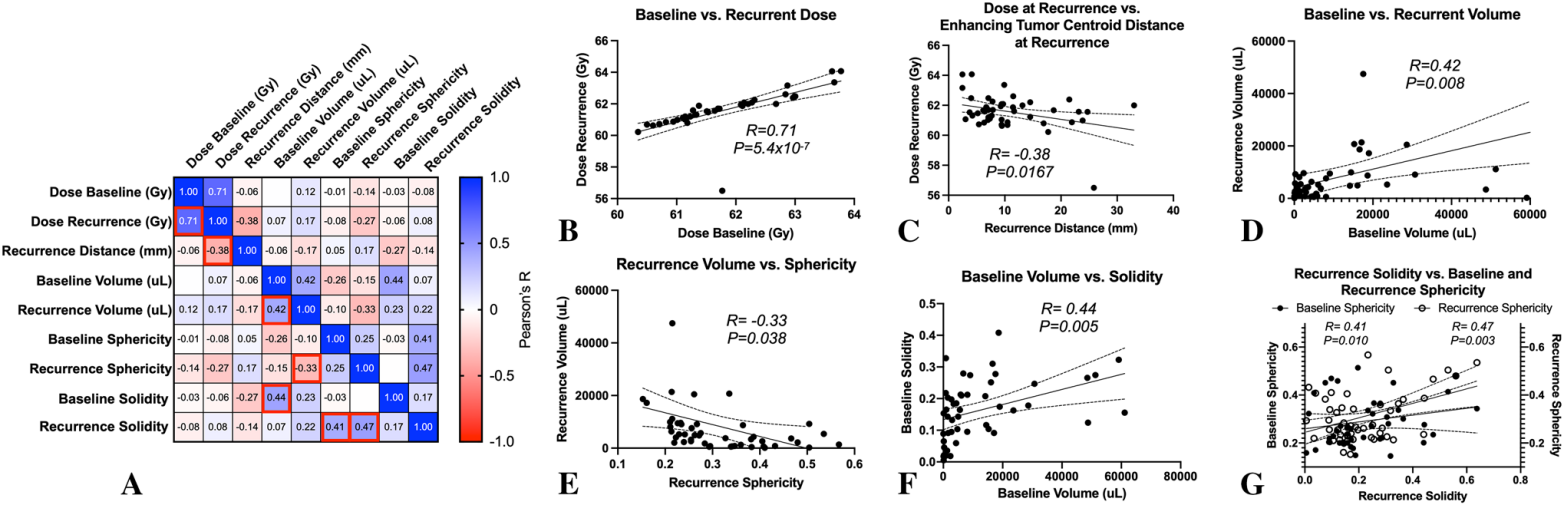
Association between dose distribution, tumor shape characteristics, and patterns of recurrence for each patient. **A** Heat map showing correlations between dose, shape, and recurrence pattern characteristics, color coded by Pearson’s correlation coefficient, *R*. Red boxes highlight associations with *P* < *0.05*. **B** Positive correlation between dose within enhancing tumor at planning with dose at the time of radiographic recurrence. **C** Negative correlation between dose within enhancing tumor at recurrence and the difference in centroid location for enhancing tumor at baseline and recurrence. **D** Positive correlation between baseline tumor enhancing volume and enhancing volume at the time of recurrence. **E** Negative correlation between the enhancing tumor volume and sphericity at the time of radiographic recurrence. **F** Positive correlation between baseline enhancing tumor volume and solidity. **G** Positive correlation between tumor sphericity at baseline and sphericity at radiographic recurrence compared with tumor solidity at recurrence

Lastly, we explored the correlation between dose distribution, tumor shape characteristics, and patterns of recurrence for each patient (Fig. 5). A heat map showing correlations between these variables are illustrated in Fig. 5A. Seven sets of variables were identified as having a significant linear correlation. These include intuitive associations, including those between baseline dose and the dose at recurrence (Fig. 5B; *R* = *0.71, P* = *5.4* × *10*^*–7*^), dose at recurrence and the difference in centroid distance at the time of radiographic recurrence (Fig. 5C; *R* = *− 0.38, P* = *0.0167*), and pre-radiation enhancing tumor volume and the volume at recurrence (Fig. 5D; *R* = *0.42, P* = *0.008*). There were also significant correlations between enhancing tumor volume and sphericity at the time of recurrence (Fig. 5E; *R* = *− 0.33, P* = *0.038*), enhancing tumor volume and solidity at baseline prior to treatment (Fig. 5F; *R* = *0.44, P* = *0.005*), and enhancing tumor solidity at recurrence and sphericity at both baseline (Fig. 5G; *R* = *0.41, P* = *0.010*) and at recurrence (*R* = *0.47, P* = *0.003*).

## Discussion

GBM recurrence remains nearly uniform [4], so improved characterization of GBM recurrence following chemoradiation remains an urgent need. The present study observed numerous associations in GBM recurrence patterns with dose distribution and lesion morphological metrics at baseline and at time of recurrence. For example, this study observed that increased change in centroid distance was associated with increased PFS. These findings are similar to previous findings by Brandes et al*.*, where they observed that recurrences distant from the RT field occurred after a longer time interval compared to those within the RT field [10], and add to the current body of literature suggesting that local recurrences within the RT field are most common in GBM and distant recurrences are more common in patients with long time to first relapse. Additionally, it is important to note that increased change in centroid distance was not found to be predictive of survival advantage in terms of PPS, which may guide clinical follow-up in GBM patients at recurrence.

Interestingly, when stratifying patients based on MGMT-status, there was a significant positive correlation between change in centroid distance and PFS for MGMT methylated patients only. It is well-known that primary and recurrent GBMs are substantially altered [16]. Kim et al. discovered that local, recurrent GBMs shared ~ 70% of gene mutations with their primary tumors while distant, recurrent GBMs shared only ~ 25% of gene mutations with their primary tumors [25], and similarly Andor et al*.* observed a trend that recurrent tumors who received RT and temozolomide had a higher number of mutations in the recurrent GBM compared to the primary tumor [17]. Furthermore, MGMT unmethylated tumors have been shown to recur within the RT field more often than MGMT methylated tumors, which was theorized to be the result of increased sensitivity of MGMT methylated GBM cells to chemoradiation within the RT field [5, 10]. In the present study, there was also a significant negative correlation between radiation dose and change in centroid distance. Although speculative, the fact that the shift in centroid distance was significantly correlated to PFS only in MGMT methylated patients may reflect greater altered tumor composition in MGMT methylated GBMs, although other factors beyond MGMT status may be present to specifically explain the correlative behavior of centroid distance and PFS in this patient subgroup.

Although there was no significant difference in the average dose within the enhancing tumor at baseline compared to recurrence, patients exhibiting tumor shrinkage following RT tended to have ~ 0.5 Gy higher average dose to the enhancing tumor compared with patients exhibiting new enhancing disease. Moreover, comparisons between voxel-wise dose distributions at baseline and at recurrence clearly revealed that some components of the tumor progress into areas of the brain that experienced subtherapeutic radiation dose, particularly in regions ≤ 40 Gy. Because of the concerns of radiation-induced neurotoxicity, the current standard in radiotherapy for glioblastomas is delivering a total dose of 60 Gy with concomitant temozolomide to the contrast-enhancing tumor. Studies with dose escalation beyond 60 Gy have shown mixed results in terms of survival benefit [7, 26, 27], and there is an increased risk of symptomatic radiation necrosis at doses of 72 Gy and 90 Gy [28]. There have also been explorations in expanding the RT target volume. For example, inclusion of the peritumoral edema in the RT target volume was found to decrease the failure rate of marginal recurrences, but no significant differences in PFS or OS were observed [29]. There has also been interest in the subventricular zone (SVZ) because of the region’s role in GBM tumorigenesis [30] and alterations following chemoradiation [31], and irradiation of the ipsilateral SVZ has been associated with improved survival for GBM patients [26, 32]. As a result, the present finding that recurrent GBMs had a larger proportion of voxels that received subtherapeutic doses compared to the primary tumor further demonstrates the neuro-oncologic need for improved management of GBM. Our findings may also support the potential benefit of combining chemoradiation with other treatments. For example, an area of growing interest has been combining immunotherapy with radiation therapy, for pre-clinical models of GBM have shown promising results [33, 34]. Further studies investigating GBM recurrence with RT dose maps in patients undergoing chemoradiation with concurrent treatments may be valuable.

To our knowledge, the present study is also the first to assess morphological features of sphericity and solidity at both baseline and recurrence. Interestingly, although there was no overall difference in sphericity at recurrence compared with baseline, tumors that progressed within 12 weeks of RT were less spherical than those that progressed after 12 weeks of RT. Previous studies on sphericity in GBMs found that reduced sphericity of both the contrast-enhancing and FLAIR hyperintense tumor of GBMs was associated with reduced OS [13] and that differences in sphericity can be one component to stratify general subtypes of GBM [35]. Moreover, in the present study, tumors had higher solidity at recurrence compared to baseline, and Chaddad et al*.* observed that increased solidity of necrotic regions of GBM was associated with reduced OS [11]. While the present study did not observe any associations between PPS and sphericity or solidity, it remains possible that the altered morphology between recurrent tumors and tumors at baseline may reflect differences in tumor severity as reflected in previous studies on GBMs at single timepoints. This is further suggested by how at baseline, larger tumor volumes were associated with higher solidity and how at radiographic recurrence, larger tumor volumes were associated with lower tumor sphericity. As a result, further investigations with a larger cohort assessing morphological differences between tumors at baseline and at recurrence, including metrics beyond sphericity and solidity, are warranted to better guide clinical decision making at GBM recurrence that combines conventional metrics such as tumor volume with advanced morphological metrics.

This study has several limitations that should be addressed. The small sample size of 48 patients may have limited the study’s capability to detect significant findings, particularly in relationships with survival outcomes with radiation dose and morphological features. For example, although MGMT methylation status is considered to be prognostic, no significant relationships were able to be found between MGMT and prognosis in this study. Because GBM is known to have genetic heterogeneity [36], further studies with a larger study cohort may be needed for understanding how other tumor genetics beyond MGMT status may be related to IDH-wild-type GBM recurrence location, dose distributions, and change in morphological features after radiation therapy. Moreover, a large majority of our study cohort (34/48 patients; 70.8%) had recurrence within 3 months of completion of RT. Considering that the majority of pseudo-progression occurs within the first 3 months after radiation [37], the present study may have benefitted from a larger study cohort with more patients who progressed after 3 months of completion of RT to better characterize patients who may have had pseudo-progression.

## Conclusion

Changes in centroid distance and morphological features of recurrent GBMs may reflect tumor alterations following chemoradiation. Dose distributions of RT at baseline may offer insights into regions of GBM recurrence. Further studies assessing morphological changes between recurrent GBMs and at baseline are warranted.

## Abbreviations

GBM: Glioblastoma
RANO: Response assessment in neuro-oncology
MGMT: *O*(6)-methylguanine-DNA methyltransferase
IMRT: Intensity modulated radiation therapy
PFS: Progression free survival
OS: Overall survival
PPS: Post progression survival
CTV: Clinical target volume
PTV: Planning target volume
GTV: Gross tumor volume
